# White matter integrity and medication response to antidepressants in major depressive disorder: a review of the literature

**DOI:** 10.3389/fpsyt.2023.1335706

**Published:** 2024-02-01

**Authors:** Giovanni Videtta, Letizia Squarcina, Cecilia Prunas, Paolo Brambilla, Giuseppe Delvecchio

**Affiliations:** ^1^Department of Biomedical Sciences for Health, University of Milan, Milan, Italy; ^2^Department of Pathophysiology and Transplantation, University of Milan, Milan, Italy; ^3^Department of Neurosciences and Mental Health, Fondazione IRCCS Ca’ Granda Ospedale Maggiore Policlinico, Milan, Italy

**Keywords:** major depressive disorder, antidepressant medications, diffusion tensor imaging, white matter, translational psychiatry

## Abstract

Major Depressive Disorder (MDD) is a severe psychiatric disorder characterized by selective impairments in mood regulation, cognition and behavior. Although it is well-known that antidepressants can effectively treat moderate to severe depression, the biochemical effects of these medications on white matter (WM) integrity are still unclear. Therefore, the aim of the study is to review the main scientific evidence on the differences in WM integrity in responders and non-responders to antidepressant medications. A record search was performed on three datasets (PubMed, Scopus and Web of Science) and ten records matched our inclusion criteria. Overall, the reviewed studies highlighted a good efficacy of antidepressants in MDD treatment. Furthermore, there were differences in WM integrity between responders and non-responders, mainly localized in cingulate cortices, hippocampus and corpus callosum, where the former group showed higher fractional anisotropy and lower axial diffusivity values. Modifications in WM integrity might be partially explained by branching and proliferation as well as neurogenesis of axonal fibers mediated by antidepressants, which in turn may have positively affected brain metabolism and increase the quantity of the serotonergic neurotransmitter within synaptic clefts. However, the reviewed studies suffer from some limitations, including the heterogeneity in treatment duration, antidepressant administration, medical posology, and psychiatric comorbidities. Therefore, future studies are needed to reduce confounding effects of antidepressant medications and to adopt longitudinal and multimodal approaches in order to better characterize the differences in WM integrity between responders and non-responders.

## 1 Introduction

Major depressive disorder (MDD) is a psychiatric disorder characterized by low mood, low self-esteem, and loss of interest in ordinary activities (1).

From a neuroimaging perspective, impairments in signal transduction caused by alteration in levels of neurotransmitters have been consistently reported in MDD (2, 3), especially in subcortical structures that are part of the limbic system and known to be involved in emotional codification and processing (4).

Among neurotransmitters, serotonin is one of the most involved due to its crucial role in mood regulation (5). Indeed, decreased levels of serotonin were observed in MDD (6), maybe due to deficits in the serotonin transporter (5-HTT) within raphe nuclei, at the level of brainstem (7), or within the amygdala and midbrain (8), as well as in the hippocampus, whose neurochemical disruption has been suggested to contribute to developing depressive and anxiety behaviors (9).

In order to rebalance the levels of serotonin in MDD, several pharmacological treatments were developed, including antidepressant medications, which aim to regulate the quantity of neurotransmitters within the brain cortex (10) and to rewire axonal fiber connections by exploiting neuroplasticity (11). Considering the individual tolerability and safety in administration, nowadays the two most used antidepressant drug classes are the Selective Serotonin Reuptake Inhibitors (SSRIs) and the Serotonin-Norepinephrine Reuptake Inhibitors (SNRIs) (12). SSRIs increase the amount of serotonin at the level of synaptic clefts by blocking the normal reuptake of the neurotransmitter (13), and their efficacy was tested against the most severe MDD diagnosis (14). The SNRIs, on the other hand, have a double action on both serotonin and noradrenaline by blocking their reuptake within the synaptic clefts and working similarly to cyclic antidepressants (15).

Beside neurotransmitter impairment, Diffusion Tensor Imaging (DTI) studies consistently reported widespread disruption within white matter (WM) tissue in MDD (16). DTI estimates the directionality of water molecules movement in each voxel and describes it with four diffusivity indices: fractional anisotropy (FA), mean diffusivity (MD), axial diffusivity (AD) and radial diffusivity (RD) (17). FA is a scalar value between 0 and 1 describing the degree of anisotropy within brain tissues; MD is an estimate of the average water movement inside the considered voxel; AD describes the diffusivity along the principal diffusion direction; and, finally, RD describes the diffusivity perpendicular to the main diffusion direction. In the field of DTI, tract-based spatial statistics (TBSS) and tractography are employed to analyze WM pathways in the brain. TBSS is a statistical method used for group-wise analysis of WM microstructure and it is based on the alignment of individual subjects’ DTI to a common space (18). On the other hand, tractography is a visualization technique that reconstructs and maps the pathways of WM tracts in the brain based on DTI data (19). In particular, in MDD structural disruptions within WM tissue are reflected by decreased FA mainly localized in the corpus callosum (CC), cingulum and uncinate fasciculus (20). Moreover, reduction of FA has been found correlating with severity of depressive symptomatology (21) and MDD duration (22), probably explaining the impairments in general cognitive functioning often observed in these patients according to anatomical disruptions (23).

Importantly, as demonstrated by several pieces of evidence, WM is very sensitive to external factors (24), such as pharmacological treatments. Indeed, drugs seem to have a great impact on axonal fibers by modifying their synaptic plasticity and structural organization (25, 26). Therefore, it is likely that during a treatment based on antidepressant medications WM undergoes neuroplasticity processes which may characterize a specific clinical outcome, as already demonstrated by functional connectivity evidence (27).

In this context, this review aims to collect evidence on WM integrity after antidepressant medications by focusing on diffusion differences of clinical outcomes, such as responders vs. non-responders and remitters vs. non-remitters, as they are paramount to describe different levels of success at the end of a pharmacological treatment (28). Responders are patients who show clinically significant improvements in their symptomatology, which is typically defined by a certain percentage reduction in symptom severity scores, often measured by standardized rating scales. On the other hand, remitters are patients who not only respond to medications but also achieve complete resolution of their symptomatology, returning to a clinical state comparable to healthy individuals.

## 2 Materials and methods

**Figure 1:**
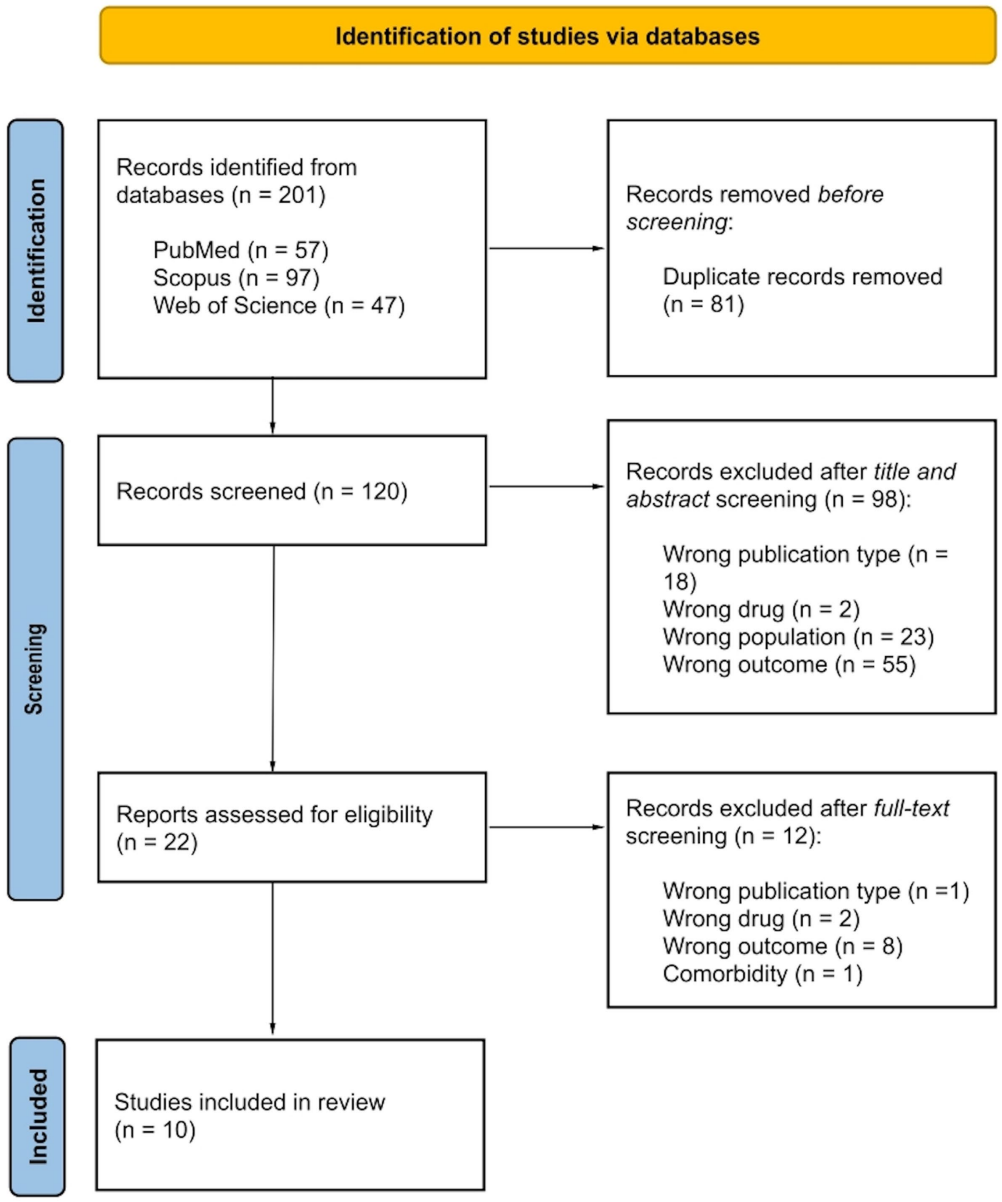
Flowchart diagram for record selection.

Record research was performed on three datasets: PubMed, Scopus and Web of Science. Combining Boolean operators the string used was the following: (Diffusion Tensor Imaging OR DTI) AND white matter AND antidepress* AND (Major Depressive Disorder OR MDD). The inclusion criteria were: (i) peer-reviewed original publication, (ii) English language, (iii) MDD diagnosis, (iv) DTI investigation of WM integrity after antidepressant medications by focusing on clinical outcomes, and (v) employment of at least one diffusion index (FA, MD, AD and/or RD) to describe WM integrity. Exclusion criteria were: (i) animal studies, (ii) current psychiatric and/or neurological comorbidities (excluding generalized anxiety disorder and social anxiety disorder), and (iii) no comparison between clinical outcomes. No limit was placed regarding antidepressant administration (e.g., type of drugs, duration of treatment, clinical efficacy) and type of clinical outcome comparisons (e.g., remitters vs. non-remitters, pre- vs. post-treatment individual conditions). Record research was performed on August 11th, 2023 with no temporal windows. In Figure 1 the record research is reported. 201 records were found, of which 120 were unique records. After title and abstract screening, 22 records were assessed for eligibility and only 10 were included in the review by matching inclusion and exclusion criteria. Table 1 provides records, extracted variables and main results.

**Table 1:** Sociodemographic and clinical variables of reviewed studies.

Study	Study design	Sample size (M/F)	Age (mean ± sd)	Clinical treatment			DTI parameters		Main results	
				Antidepressant medications	Dosage	Length of therapy	Acquisition (Tesla, direction, voxel—mm^3^)	Indices	Clinical outcome	WM integrity
Alexopoulos et al. (29)	Cross-sectional	MDD: 48 (ns/ns)	70.2 ± 5.8	Escitalopram	10 mg/dd	12 wk	1.5 T, 8, 5 × 5 × 5	FA	•Remitters (25) •Non-remitters (23)	↑FA ACC, DLPFC, genu (CC), hippocampus, PCC, insula.
Davis et al. (30)	Cross-sectional	MDD: 165 (61/104)	35.7 ± 12.5	Escitalopram	10-20 mg/dd	•2 wk. •8 wk	•3.0 T, 30, 2.5 × 2.5 × 2.5 •3.0 T, 31, 2.5 × 2.5 × 2.5	•FA •MD •AD •RD	•Responders (80) •Non-responders (85)	↓AD bilateral cerebral peduncle, L PTR, R CgC, bilateral CgH, L EC.
Dong et al. (31)	Cross-sectional	MDD: 127 (58/69)	35.3 ± 9.1	•SSRI •SNRI	ns	24 wk	3.0 T, 32, 3 × 3 × 3	FA	•Remitters (62) •Non-remitters (65)	NS FA.
Hoogenboom et al. (32)	Cross-sectional	MDD: 92 (34/58)	46.5 ± 14.6	•SSRI •NDRI •SNRI •TCA •Lithium •NaSSA •SARI	ns	12 mo	•1.5 T, 7, 6 x 6 x 6 •1.5 T, 16, 6 × 6 × 6 •1.5 T, 22, 6 × 6 × 6 •1.5 T, 23, 6 × 6 × 6 •1.5 T, 28, 6 × 6 × 6 •1.5 T, 35, 6 × 6 × 6 •1.5 T, 39, 6 × 6 × 6 •1.5 T, 70, 6 x 6 x 6	FA	•Remitters (63) •Non-remitters (29)	↑FA medial fornix.
Korgaonkar et al. (33)	Cross-sectional	MDD: 80 (40/40)	33.8 ± 13.1	•Escitalopram •Sertraline •Venlafaxine-XR	•10-20 mg/dd (Escitalopram) •50-200 mg/dd (Sertraline) •75-225 mg/dd (Venlafaxine-XR)	8 wk	3.0 T, 42, 2.5 × 2.5 × 2.5	FA	•Remitters (37) •Non-remitters (43)	↑FA CgC. ↓FA stria terminalis.
Pillai et al. (34)	Cross-sectional	MDD: 144 (54/90)	37.2 ± 13.7	Sertraline	200 mg/dd (maximum dose)	8 wk	•ns, 64, 2.5 × 2.5 × 2.5 •ns, 64, 1.9 × 1.9 × 1.9	•FA •MD •AD •RD	•Remitters (53) •Non-remitters (91)	↓FA WM tracts raphe nucleus-bilateral amygdala.
Seiger et al. (35)	Longitudinal	MDD: 33 (17/16)	29.2 ± 9.6	Citalopram	8 mg/dd	2 dd	3.0 T, 30, 2 × 2 × 2	•FA •MD •AD •RD	na	↓MD L ACR, L EC, CC. ↓AD L EC, genu (CC), splenium (CC), inf frontal blade. ↓RD L ACR, sup frontal blade.
Tatham et al. (36)* *(the study employed a MDD subgroup who was administered quetiapine XR)	Cross-sectional	MDD: 24 (ns/ ns)	ns ± ns	Citalopram	20 mg/dd (Citalopram)	8 wk	3.0 T, 12, 3 × 3 × 3	FA	•Responders (9) •Remitters (8) •Non-responders (7)	NS FA.
Taylor et al. (37)	Cross-sectional	MDD: 74 (40/34)	68.1 ± 6.8	Sertraline	50-200 mg/dd (maximum dose)	12 wk	1.5 T, 6, 3 x 3 × 3	FA	•Remitters (37) •Non-remitters (37)	↓FA R ACC.
Vieira et al. (38)	Cross-sectional	MDD: 20 (6/14)	37.7 ± 12.2	Paroxetine	20 mg/dd	12 wk	1.5 T, 30, 2 x 2 × 2	•FA •MD •AD •RD	•Responders (12) •Non-responders (8)	↑FA forceps minor, bilateral SLF, L IFOF. ↓RD L SLF.

↑, increase; ↓, decrease; ACC, Anterior Cingulate Cortex; ACR, Anterior Corona Radiata; AD, Axial Diffusivity; CC, Corpus Callosum; CgC, Cingulum Cingulate; CgH, Cingulum Hippocampus; dd, Day(s); DLPFC, Dorsolateral Prefrontal Cortex; DTI, Diffusion Tensor Imaging; EC, External Capsule; F, Females; FA, Fractional Anisotropy; IFOF, Inferior Frontal Occipital Fasciculus; inf, Inferior; L, Left; M, Males; MD, Mean Diffusivity; mg, Milligrams; MDD, Major Depressive Disorder; mo, Month(s); na, Not Applicable (not recorded variable); NaSSA, Noradrenergic And Specific Serotonergic Antidepressant; NDRI, Norepinephrine and Dopamine Reuptake Inhibitors; NS, Not Significant; ns, Not Specified (recorded but not reported variable); PCC, Posterior Cingulate Cortex; PTR, Posterior Thalamic Radiation; R, Right; RD, Radial Diffusivity; SARI, Serotonin Antagonist and Reuptake Inhibitors; sd, Standard Deviation; SLF, Superior Longitudinal Fasciculus; SNRI, Serotonin-Norepinephrine Reuptake Inhibitor; SSRI, Selective Serotonin Reuptake Inhibitors; sup, Superior; TCA, Tricyclic Antidepressant; WM, White Matter; wk, Weeks.

## 3 Results

All studies employed SSRIs and SNRIs for MDD treatment. Beside SSRIs and SNRIs, Hoogenboom et al. (32) investigated the combined effects of several antidepressants, including norepinephrine and dopamine reuptake inhibitors, tricyclic antidepressants, lithium, noradrenergic and specific serotonergic antidepressants, serotonin antagonist and reuptake inhibitors. The majority of the studies adopted a pharmacological treatment lasting in total between 8 and 12 weeks, except for three studies that considered either a more extended (24 weeks and 12 months) (31, 32) or shorter (2 days) (35) temporal window. Out of ten studies, only four investigated MD, AD and RD other than FA (30, 34, 35, 38). Finally, with regards to DTI analysis, only five studies employed TBSS (31, 33, 35, 36, 38), one tractography (34), and the remaining ones a voxel-based approach (29, 30, 32, 37).

### 3.1 Single-drug treatment

Six studies administered a single-drug treatment and investigated WM integrity of MDD patients by considering both remission and response as clinical outcomes (29, 30, 34, 35, 37, 38).

Alexopoulos et al. (29) administered 10 milligrams per day (mg/dd) of escitalopram to 48 MDD elderly patients for 12 weeks. At the end of treatment, 25 patients were remitters and 23 were non-remitters. The authors found that compared to non-remitters, remitters showed higher FA in several WM regions, including anterior cingulate cortex (ACC) and posterior cingulate cortex, dorsolateral prefrontal cortex, genu of CC, hippocampus, and insula.

Similarly, Davis et al. (30) investigated the effect of escitalopram (10-20 mg/dd) on WM and clinical outcome in 165 MDD patients after 2 and 8 weeks of treatment. Interestingly, the authors found that the most significant differences between 85 responders and 80 non-responders were observed after 8 weeks of treatment. Specifically, compared to non-responders, responders had lower AD in bilateral cerebral peduncle, left posterior thalamic radiation, right cingulum cingulate (CgC), bilateral cingulum hippocampus, and left external capsule (EC). No significant differences were observed for FA, MD and RD.

On the other hand, Pillai et al. (34) explored the effect of sertraline on WM integrity and clinical outcome in 144 MDD patients from Establishing Moderators and Biosignatures of Antidepressant Response in Clinical Care (EMBARC) dataset. After an 8 weeks treatment with a maximum dosage of 200 mg/dd, 53 patients were remitters and 91 were non-remitters. Compared to non-remitters, remitters showed lower FA in WM tracts connecting the raphe nucleus and bilateral amygdala without, though, showing any significant difference in MD, AD and RD.

These results were only partially confirmed by Taylor et al. (37), who designed a similar study with 74 MDD patients by focusing only on FA and following 12 weeks of treatment based on sertraline (50–200 mg/dd). Among 37 remitters, lower FA was found within the right ACC in comparison with the 37 non-remitters.

Finally, Seiger et al. (35) and Vieira et al. (38) investigated the effects of citalopram and paroxetine, respectively, on WM integrity in MDD patients. Seiger et al. (35) analyzed the short-term effects of citalopram according to a 2-days treatment with a dosage of 8 mg/dd. In 33 MDD patients the authors evaluated FA, MD, AD and RD pre- and post-treatment and found lower MD in left anterior corona radiata (ACR), left EC and CC; lower AD in left EC, genu and splenium of CC, inferior frontal blade; lower RD in left ACR and superior frontal blade. Finally, Vieira et al. (38) recruited 20 MDD patients who underwent paroxetine treatment for 12 weeks. The WM integrity was compared between 12 responders and 8 non-responders. In responders, higher FA was localized in forceps minor, bilateral superior longitudinal fasciculus (SLF) and left inferior fronto-occipital fasciculus whereas reduced RD was localized in left SLF.

### 3.2 Multiple-drug treatment

Four studies employed antidepressant medications based on administration of multiple drugs in order to investigate the clinical outcomes related to WM integrity among MDD patients (31–33, 36).

In a sample of 127 MDD patients, Dong et al. (31) investigated FA differences between remitters and non-remitters after administration of several SSRIs and SNRIs for 24 weeks. At the end of treatment, patients who achieved remission were 62, while those who did not were 65. However, no significant differences in FA were observed between remitters and non-remitters.

In contrast, Hoogenboom et al. (32) carried out a legacy-data study on 92 MDD patients. Authors retrieved clinical records of patients who had been recruited from 1999 to 2009 and had been administered different antidepressants for a period of almost 24 months. The only significant result found by authors was the higher FA in medial fornix in 63 remitters compared to 29 non-remitters.

Also, Korgaonkar et al. (33) administered two SSRIs, escitalopram and sertraline, and an SNRI, venlafaxine-XR, to 80 MDD patients for 8 weeks. The dosage was different for each drug: 10-20 mg/dd, for escitalopram; 50-200 mg/dd, for sertraline; 75-225 mg/dd, for venlafaxine-XR. Similar to Dong et al. (31) and Hoogenboom et al. (32), the authors investigated only FA in 37 remitters and 43 remitters and reported that the former group had higher FA in CgC and lower FA in stria terminalis compared to the latter group.

Finally, Tatham et al. (36) administered citalopram (20 mg/dd) to 24 MDD patients, at the end of which the authors examined WM integrity through FA. Between 9 responders, 7 non-responders and 8 remitters no statistical difference was detected in FA values.

## 4 Discussion

The present study reviewed the literature on WM integrity after antidepressant medications by comparing the clinical outcomes of MDD patients. Overall, the results showed that MDD patients who achieved remission or responded to the pharmacological treatment, showed a difference in DTI indices in respect to non-remitters and non-responders, mainly localized in cingulate cortices, hippocampus and CC.

Overall, considering the efficacy of antidepressant medications, almost half of reviewed studies demonstrated a good rate of clinical outcome (29, 32, 36–38), in line with previous evidence reported by the literature (39). However, almost all the reviewed studies employed a cross-sectional design and, therefore, it is not possible to state whether the differences in WM integrity are due to the effect of medications or were already in place before administering antidepressants. Only Seiger et al. (35) designed a longitudinal study showing partial recovery effects on WM integrity before and after pharmacological treatment, ultimately suggesting that antidepressants may have a normalizing effect on axonal fiber organization.

As described by the reviewed studies the main effects of antidepressant medications were found within cingulate cortices, where, in line with literature, an overall increase of FA occurs in MDD patients at the end of pharmacological treatment (40). Specifically, the reviewed studies found a difference in WM integrity in the cingulum between remitters and non-remitters (29, 33), and between responders and non-responders (30), with the formers showing higher FA and lower AD, respectively. The cingulum belongs to the limbic system and creates anatomical connections with thalamus, hypothalamus and brainstem nuclei (41). Its functional role is related to emotional codification which characterizes depressive symptomatology (42) and, therefore, it is not surprising that in MDD it is one of the most disrupted structures (43). A lower FA in the cingulum might be associated with dysfunctions in connectivity patterns (44) due to either a reduction (45) or a demyelination (46) of axonal fibers. Moreover, abnormal connectivity in cingulate cortices determines an altered activation in frontal regions (47, 48), which correlated with the severity of MDD (49, 50). The specific effects of antidepressant medications on cingulate cortices might be explained by structural modifications within axonal fibers related to proliferation and branching processes (51). This would be coherent to higher WM integrity in responders and may reflect both an improvement in brain metabolism and an increase of serotonergic neurotransmitter within synaptic clefts (52, 53).

Similarly, at the end of SSRIs treatment two reviewed studies (29, 30) also reported higher FA and lower AD within the hippocampus in remitters and responders compared to non-remitters and non-responders. Hippocampus is a key region known to be involved in the pathophysiology of MDD whose abnormal neurogenesis has always been identified as a distinctive trait of depressive symptomatology (54). Hippocampal neurogenesis involves the normal growth of axonal fibers (55) and has been revealed to be sensitive to stress (56) and brain-induced neurotrophic effects (57). Delay or stop of neurogenesis within hippocampal cortices has important consequences on brain metabolism (58), volumetric dimensions (59) and synaptic organization (60). Therefore, by considering hippocampal neurogenesis as clinical target for MDD treatment (54), the higher FA observed in remitters and responders after antidepressant medications might be due to the reactivation of axonal fiber growth which may reverse the atrophy process (61) and up-regulate the neurotrophin signaling pathways (62). In other words, pharmacological treatment based on SSRIs seem to restore 5-HTT deficits (63, 64) by mediating the pro-proliferate effect of antidepressant response (65) and guaranteeing prolonged outcomes even after the total attenuation of depressive symptomatology (66).

Finally, some reviewed studies (29, 35, 38) found higher FA and lower AD in CC between responders and non-responders at the end of antidepressant medications. The CC has been consistently found disrupted in MDD patients, especially the genu, where a decrease of FA, probably connected with a reduction of axonal fibers (67), has been largely reported (68, 69). According to WM neuroanatomy, the axonal fibers originating from genu constitute forceps minor, a well-known clinical biomarker for MDD treatment (38), and reach the frontal regions (70). Therefore, decreased FA in genu of MDD patients may be linked to functional deactivation of those networks which are involved in emotional codification within the frontal cortex (71). As for cingulate cortices, also for CC, the effects of antidepressant medications on WM integrity might be explained by axonal proliferation and branching, which in turn determine higher FA in responders (72).

However, in order to correctly interpret these results, some study limitations should be considered. First, reviewed studies did not employ specific analysis to investigate the impact of each antidepressant in multiple-drug treatments. Second, the effects of different medical dosage on WM integrity were not quantified. Third, the heterogeneity of length of treatment and the extraction of only one DTI index (FA) may not allow a clear interpretation on the effect of antidepressant medications on WM tissue. Fourth, some of the reviewed studies often recruited individuals with comorbid anxiety disorders which did not allow to generalize the observed findings to just MDD patients. Fifth, another limitation associated with inclusion and exclusion criteria can be identified in the choice of selecting only English language publications which could provide a biased assessment of a topic, and can lead to biased results in literature reviews. Finally, we have not conducted a full systematic review therefore our work has intrinsic limits mostly related to deep search (e.g.: bibliography screening) and critical evaluation (e.g.: quality bias assessment) of papers as well as retrieval of missing variables of interest.

In conclusion, from the reviewed studies it emerged that at the end of pharmacological treatment MDD patients who achieved remission or responded to antidepressant medications, showed higher WM integrity mainly localized in cingulate cortices, hippocampus and CC. This would ultimately suggest that antidepressants may indeed have neurotrophic effects on selective brain regions, which may be therefore considered putative clinical biomarkers for MDD treatment. However, future research is warranted for at least two reasons. First, it is necessary to consider the confounding effects of external factors related to antidepressant medications and of overlapping comorbidities related to anxiety disorders. Second, it becomes essential to employ more refined neuroimaging techniques to better characterize neurobiological processes underlying the modifications in WM integrity. This would increase our understanding on the etiology of MDD and will allow the identification of more personalized clinical treatments for depressive patients.

## Author contributions

GV: Writing – original draft. LS: Writing – review & editing. CP: Writing – review & editing. PB: Supervision, Writing – review & editing. GD: Supervision, Writing – review & editing.
